# Sono-processes: Emerging systems and their applicability within the (bio-)medical field

**DOI:** 10.1016/j.ultsonch.2023.106630

**Published:** 2023-10-04

**Authors:** Clio Siebenmorgen, Albert Poortinga, Patrick van Rijn

**Affiliations:** a University of Groningen, University Medical Center Groningen, Department of Biomedical Engineering-FB40, Deusinglaan 1, Groningen 9713 AV, The Netherlands; b Technical University Eindhoven, Department of Mechanical Engineering, Gemini Zuid, de Zaale, Eindhoven 5600 MB, The Netherlands

**Keywords:** Sonochemistry, Ultrasound, Mechanophore, Sonoelectrochemistry, Sonocatalysis, Sonocrystallization

## Abstract

Sonochemistry, although established in various fields, is still an emerging field finding new effects of ultrasound on chemical systems and are of particular interest for the biomedical field. This interdisciplinary area of research explores the use of acoustic waves with frequencies ranging from 20 kHz to 1 MHz to induce physical and chemical changes. By subjecting liquids to ultrasonic waves, sonochemistry has demonstrated the ability to accelerate reaction rates, alter chemical reaction pathways, and change physical properties of the system while operating under mild reaction conditions. It has found its way into diverse industries including food processing, pharmaceuticals, material science, and environmental remediation. This review provides an overview of the principles, advancements, and applications of sonochemistry with a particular focus on the domain of (bio-)medicine. Despite the numerous benefits sonochemistry has to offer, most of the research in the (bio-)medical field remains in the laboratory stage. Translation of these systems into clinical practice is complex as parameters used for medical ultrasound are limited and toxic side effects must be minimized in order to meet regulatory approval. However, directing attention towards the applicability of the system in clinical practice from the early stages of research holds significant potential to further amplify the role of sonochemistry in clinical applications.

## Highlights

• Sonochemistry has demonstrated the ability alter chemical reactions and change physical properties under mild reaction conditions.
• Ultrasound has found its way into diverse industries but limitedly in the biomedical field.
• In the (bio-)medical field ultrasonic cleaning and imaging are most established but use in sonodynamic therapy (SDT) and drug delivery systems is emerging.
• To pave the way for sonochemically application-driven advancements, various fundamental aspects need to be addressed.

## 1 Introduction

**Fig. 1:**
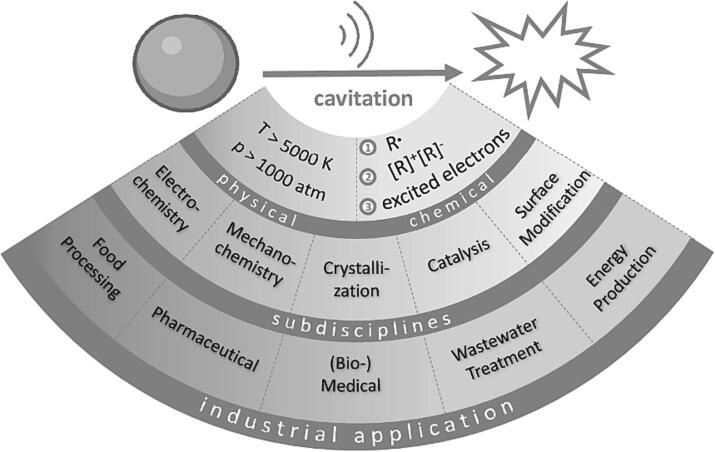
Schematic illustration of sonochemistry including the induced chemical and physical changes of the reaction, its branches of chemistry and fields of industries in which sonochemistry shows promising applications.

The earliest documented evidence of Ultrasound (US) waves, which refer to sound waves with frequencies beyond the upper limit of human hearing, starting from 20 kHz, dates back to 1794 when Spallanzani conducted investigations into the navigation behavior of bats in darkness [1]. Around 120 years later, in 1917, Langevin and Chilowsky introduced the first practical application of US, known as the 'Hydrophone', which facilitated the detection of submarines using ultrasonic waves [2], [3]. Since then, US has found its way into numerous industries, with medical diagnostics being one of its most prominent applications. However, despite its extensive utilization, there has been a lack of comprehensive understanding regarding the underlying theory of ultrasonic waves and their potential use for chemical reactions. This aspect was largely overlooked until the late 20th century when research in sonochemistry began to gain recognition and attention [4], [5], [6]. Sonochemistry, focuses on investigating the chemical and physical effects of US in guiding chemical reactions (Fig. 1). However, to effectively utilize sonochemistry, a comprehensive understanding of the underlying principles of US is vital in order to predict its impact on chemical reactions. Ultrasonic waves are generated using a transducer, which converts electrical energy into sound waves via the piezoelectric effect. Within the transducer, piezoelectric crystals undergo deformation when an electric field is applied, resulting in mechanical vibrations and the production of ultrasonic waves [7]. In the context of sonochemistry, the common frequency range is between 20 kHz and 1 MHz [8]. These waves propagate through mostly liquid medium and generate high- and low-pressure regions. As a result, four distinct phenomena arise: acoustic cavitation, microstreaming, vibration and acoustic scattering [9]. Among these, the most important phenomenon for the field of sonochemistry is acoustic cavitation, which defines the formation, growth, oscillation and collapse of microbubbles (MBs). The collapse of these bubbles generates localized regions known as ‘hot spots’, characterized by extreme conditions with temperatures surpassing 5000 K and pressures exceeding 1000 atm [10]. The conditions generated during cavitation have the ability to promote chemical reactions and enhance mass transfer rates. Furthermore, they induce changes in the physical properties of the liquid, such as viscosity and solubility, and promote the dispersion of reactants. The latter is especially beneficial for the dispersion and homogenization of for example oil-in-water emulsions [11], [12], [13]. Furthermore, hot spots are able to generate reactive species, including radical formation, ions, and excited molecules [14]. These species play a crucial role in initiating or expediting chemical reactions that would otherwise proceed slowly or not occur at all under normal conditions [15], [16], [17]. Consequently, US presents itself as a promising tool in chemical reactions, offering potential in altering known reactions. Furthermore, milder reaction conditions and improving the overall energy efficiency makes ultrasonic waves a good candidate in elaborating new pathways in green chemistry. In this review, we outline the key domains of sonochemistry and underscore their significant discoveries. Additionally, we demonstrate the capability of US to modify or enhance chemical reactions, showcasing notable examples of sonochemistry that have been implemented across various industries.

## 2 Fields of sonochemistry

So far, the physical background of US and the underlying physiochemical principle of sonochemistry have been discussed. The next part will discuss different sub-disciplines of sonochemistry including examples of fundamental findings and the most recent developments.

### 2.1 Sonoelectrochemistry

Sonoelectrochemistry is the subdiscipline that uses US to enhance the electron transfer reaction during the anodic oxidation and cathodic reduction reaction. Sonoelectrochemistry was discovered in the early 20th century, when ultrasonic waves accelerated the reaction rate and lowered the required voltage during water electrolysis utilizing a platinum electrode [18]. Since then sonoelectrochemistry has resulted in a continuous progress in many different applications of chemistry, such as electrodeposition, synthesis of metal alloys, sonoelectroanalysis, sustainable chemistry, and organic sonochemistry [19], [20], [21], [22], [23]. Hereby, the most interesting possibilities are highlighted that US offers when combined with electrochemical processes.

#### 2.1.1 Electrodeposition

Electroplating or electrodeposition has been the first application of sonoelectrochemistry. In this way, a wide range of nanostructures of coated materials can be created, such as nanoparticles (NPs), nanofilms, nanosheets, nanowires, and carbon nanotubes [21], [24], [25], [26], [27]. Nowadays, it is a widely used tool to coat various metallic deposits such as silver or gold nanorods, but it is also used for bioactive coatings, such as CdSe films [28], [29], [30].

Li et al. investigated the difference between electrodeposition of Ni/diamond composite coatings under mechanical agitation and simultaneous mechanical and ultrasonic agitation [31]. For ultrasonic agitation, an external US bath was used with a low frequency of 40 kHz and an acoustic power of 300 W.

**Fig. 2:**
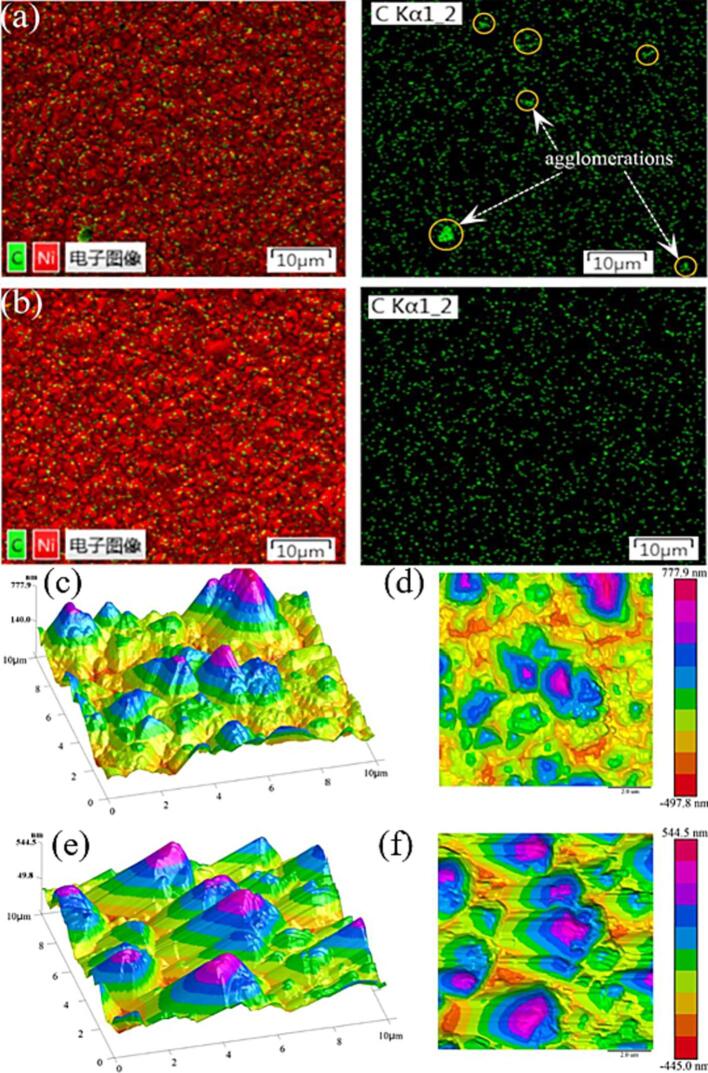
Electrodeposition of Ni/diamond coatings (a) elemental distribution of electrodeposited coating under magnetic stirring and (b) ultrasonic agitation, surface topography of electrodeposited coating under magnetic stirring (c,d) and ultrasonic agitation (e,f). Adjusted from reference [31] with permission of Elsevier.

US-assisted electrodeposition showed many advantages over magnetic stirring. The average roughness of the coating decreased when US was combined with magnetic stirring during the electrodeposition process. In particular, an increase in the uniform distribution of the particles when ultrasonicated was obtained (Fig. 2b), whereas solely magnetic stirring created agglomerations of the particles (Fig. 2a). This is an advantageous feature as it makes the coating more corrosion resistant.

Furthermore, ultrasonic agitation led to a more consistent growth orientation of Ni crystals (Fig. 2e and f) when compared to the electrodeposited coating under magnetic stirring (Fig. 2c and d). In this way also the preferred orientation plane of the Ni/diamond coatings increased.

Besides the work by Li et al., it has been shown that composition, morphology, and surface topography can alter when using US-assisted electrodeposition [20], [32]. A more detailed overview of the effects of process variables of electrodeposited coatings using US can be obtained from the recently published review from Costa et al. [26]. In particular, the acceleration of the underlying chemical process is a major advantage of US-assisted electrochemistry. Furthermore, acoustic cavitation prevents or reverses agglomeration, which promotes homogenous distributions of reactants and ultimately leads to uniform surfaces [33]. Especially uniformity of coatings plays a great role when it comes to corrosion resistance. Furthermore, the operation conditions of the ultrasonic device, such as the frequency and duration used, can influence the resulting coating properties. From these examples, it can be concluded that US offers promising possibilities to tailor the desired physiochemical properties by varying the reaction conditions. As mentioned before, sonoelectrochemistry is not only used for electrodeposition. US also plays a role in electroanalysis/electrochemical analysis since it can be used to analyze metals such as Ag, and Ni, organic ions such as NO_2_–, and organic compounds such as aminosalicylic acid and nitrofurazone [21], [30], [34], [35], [36].

#### 2.1.2 Sustainable Sonoelectrochemistry: From green chemistry to hydrogen production

Sonoelectrochemistry is also used for the degradation of organic pollutants such as amaranth dyes, formic acid, and herbicides [37], [38], [39], [40]. It has also been shown that US-assisted electrochemistry offers possibilities when it comes to hydrogen production [41]. Nowadays, the largest source for global hydrogen production comes from natural gas, mainly methane, which results in a high impact on CO_2_ emissions [42]. Therefore, technologies that enable the production of environmentally friendly, carbon-free hydrogen have become an attractive field in research. For instance, carbon-free hydrogen production can be achieved by electrochemical water splitting [43]. However, for this process high energy consumptions are required. In these cases, US assisted electrolysis is promising, as it increases the overall energy efficiency [44]. The most prominent mechanism to reduce the energy consumption, is the cavitation process of MBs as it leads to highly reactive species (H·, OH·, H_2_O_2_) in aqueous medium. This helps the electrolysis process to split water into hydrogen and oxygen. In addition, disengagement of bubbles from the electrodes can be achieved when electrolytically splitting water in the presence of ultrasonic waves [41].

#### 2.1.3 Organic sonoelectrosynthesis

Another significant aspect of sonoelectrosynthesis involves the use of US in organic electrochemistry. It has been shown in several US-assisted organic electrosynthesis reactions that ultrasonication can significantly reduce waste outcomes. Furthermore, new, shorter synthetic routes, milder reaction rates, increased yields, and regiospecific products could be obtained [23], [45]. This makes organic sonoelectrosynthesis an interesting field for a sustainable approach.

**Fig. 3:**
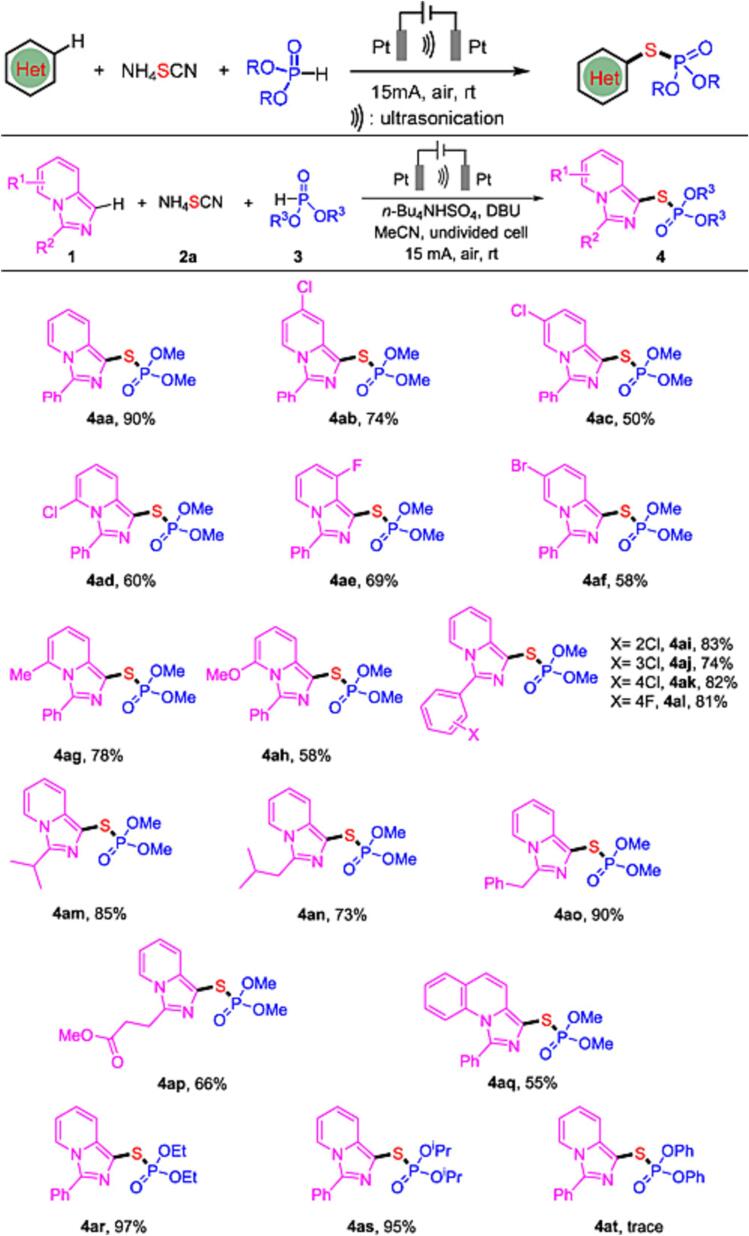
Regioselective C-H Phosphorothiolation of (Hetero-)arenes enabled by sonoelectrochemistry. Adjusted from reference [46] with permission from American Chemical Society.

Meng et al. showed that sonoelectrochemistry enabled a regioselective synthesis of C-H phos-phorothiolation of (hetero)arenes, where thiocyanate was used as the S source [46]. Usually, S-(hetero)aryl phosphorothioates are synthesized, using external oxidants or metal catalysts to mediate the reaction [47], [48]. Meng et al., however, managed to perform phosphorothiolation of (hetero)arenes under mild conditions preventing the use of external oxidants and transition metals. Furthermore, pre-functionalization of aryl precursors was not necessary. Moreover, combining electrooxidation und ultrasonication accelerated the overall reaction, showing a new green synthesis pathway. The reactions were performed in an undivided cell with platinum electrodes under a constant current of 15 mA, together with an ultrasonic device, and low frequency waves of 40 kHz at a power of 50 W at room temperature conditions were applied. Meng et al. were the first to report the successful combination of thiocyanate and dimethyl phosphite as a replacement of thiophosphates, allowing widening of the substrate scope. This synthesis has been demonstrated with a variety of imidazo[1,5-*a*]pyridines (Fig. 3). Incorporation of either electron-withdrawing or electron-donating groups on the pyridine ring gave moderate yields. Also, a variety of phosphates was investigated. The highest yield of phosphothiolated product was achieved when using diethyl phosphite and diisopropyl phosphite with yields of 97 % and 95 % respectively. In general, sonoelectrosynthesis is recognized for its increased efficiency, environmental friendliness, and potential to promote selective reactions. However, determining optimal sonication parameters such as frequency, intensity, and duration of applied ultrasonic waves for specific reactions can be time consuming. Furthermore, studies often neglect to assess how these parameters affect the corresponding reaction.

### 2.2 US induced mechanochemistry

Mechanochemistry is a subdiscipline in chemistry in which chemical reactions are stimulated by mechanical activation [49]. Thus, intramolecular bonds are broken, which induce further chemical reactions. Mechanical activation can be achieved by grinding, milling, shearing, and stretching [50], [51], [52], [53]. However, large forces are often required to trigger bond breakage for which sonication is a simple tool. Cavitation in liquid medium releases high energy, leading to bond breakages.

#### 2.2.1 Mechanophores

**Fig. 4:**
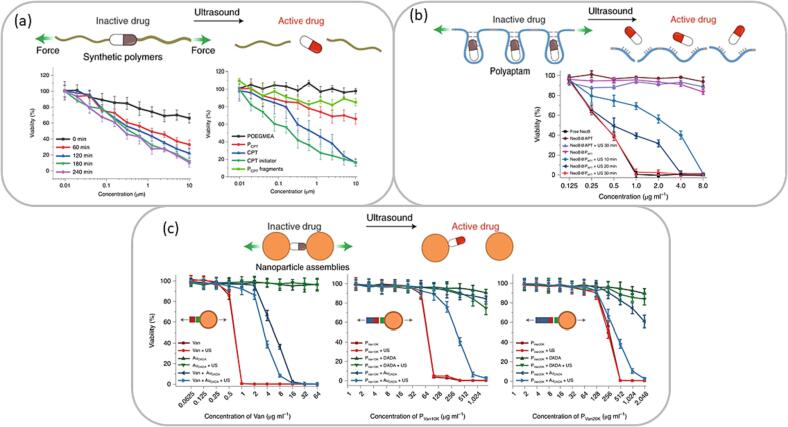
Three different approaches for specific drug activation using US on the basis of mechanophores. (a) Incorporation of inactive drug in synthetic polymer-based system, (b) Incorporation of inactive drug in RNA polyaptamer, (c) Supramolecular Au-NP assembly with inactive drug on the surface. Adjusted from reference [58] with permission from Springer Nature.

Mechanophores, which are mechanically responsive molecules, consist of predetermined breaking sites, where mechanochemically labile bonds are incorporated into the molecular framework [54]. In this way, tailoring the desired breaking sites of molecules enables the design of highly controllable, and alternative reaction pathways [55]. The reactivity of mechanophores is not only dependent on the external mechanical force, but also on the regiochemistry of the molecule [56], and electronic factors, such as isotope substitution [57], play a role. This section will focus on US activated mechanochemistry and will highlight intrinsic developments from past years. Huo et al. were able to design three pathways for drug activation using different chemical frameworks as mechanophores (see Fig. 4), which were all exposed to low frequency US using a wavelength of 20 kHz [58]. Their first approach was based on a synthetic polymer in which an inactive drug was incorporated in the framework. The breaking site of the polymer consisted of a disulfide bond. Upon irradiation, the disulfide bond reacted to thiols, which induced an intramolecular *5-exo-trig* cyclization, resulting in the activation of camptothecin, an anticancer drug (Fig. 4a). Next, an antibiotic was incorporated in RNA polyaptamers via non-covalent interactions. US destroyed these interactions, which, consequently, activated the incorporated antibiotic (Fig. 4b). Their last approach focused on supramolecular bindings using gold NPs (Fig. 4c). First, they synthesized small gold NPs decorated with the antibiotic vancomycin. Next, bigger gold NPs were pre-functionalized with the H-bond complementary target sequence of vancomycin. Both gold NPs underwent supramolecular assembly, which was destroyed once exposed to ultrasonic waves, ultimately leading to the activation of vancomycin.

This work shows three entirely different approaches to selectively activate drugs upon ultrasonication. It also demonstrates the importance of designing the breaking sites of mechanophores to achieve a highly controllable mechanosensitive response. This research is opening novel avenues for inducing drug activation through ultrasonic waves. However, translating these findings into practical applications within the (bio-)medical field, presents challenges as US waves with a frequency of 20 kHz were used. The clinical utility range of US is at much lower power levels, namely in the frequency range of 1–12 MHz. Using high power, low frequency US waves significantly increases biological effects, such as endothelial damage, DNA scattering, loss of lactate dehydrogenase, and chromosomal damage [59], [60], [61]. Therefore, future research should further elaborate on the clinical feasibility of drug activation using US frequencies in the medical applicable range. Numerous articles report controlled drug activation upon ultrasonication based on either covalent bonds, such as disulfide scission [62], [63], or non-covalent supra-molecular interactions [64]. A notable example of the latter is developed by Küng et al., in which drugs were encapsulated in polymer-grafted supramolecular cages [65]. Ultrasonication triggered the disassembly of the cage, resulting in drug activation. A detailed overview of current US-induced drug activation systems is described by Yildiz et al. [66]. Sonomechanochemistry is not only useful for precise drug activation, but also plays an important role in polymer chemistry [67].

#### 2.2.2 Polymer mechanochemistry

When molecular frameworks of polymers do not include well-defined breaking sites, exposure to US leads to unpredictable cleavage, where multiple scission events can occur on a single chain [54], [68]. Ultrasonic waves and the resulting cavitation process leads to stress on the polymer backbone [54]. As a consequence, chain scission occurs, mostly near the midpoint of the polymer backbone, where molecular forces are highest [55]. To direct controlled cleavage, understanding of prescient breaking sites is indispensable. Several parameters such as the frequency, intensity, and duration of ultrasonic waves, but also the temperature, viscosity, and gas solubility of the reaction medium affect the cleavage of breaking sites [69], [70]. Controlling the breaking site of polymers can also be achieved by incorporating mechanophore units in the polymers backbone [68].

Klein et al. were able to calculate mechanochemical activity including the breaking sites of mechanophores by computational chemistry [71]. The Constrained Geometries Simulate External Force (CoGEF) method simulates mechanical forces on molecular frameworks to predict mechanochemical activity including the breaking sites of mechanophores [72]. Furthermore, they also showed that computational predictions were in agreement with experimental results. Their work offers promising opportunities to deepen the understanding of mechanophores and enables the design of new mechanosensitive molecules with controlled breaking sites. Controlled incorporation of breaking sites was first reported by Moore’s group in 2005, where poly(ethylene glycol) was functionalized with one azo group in the polymer backbone [73]. Upon ultrasonication, scission occurred at the weak azo groups, resulting in low polydispersity of fragments. Since then, many stress-responsive polymers have been discovered and investigations have been made to further tailor site specific bond cleavage of polymer backbones. Ramirez et al. reported controlled crosslinking activation upon ultrasonication by functionalizing polybutadiene with the mechanophore dibromocyclopropane [74]. When applying destructive shear forces, dibromocyclo-propane reacts to allylic bromides, which ultimately crosslink to carboxylates via nucleophilic substitution. In this way, ultrasonic exposure to the polymer network resulted in strength properties that were magnitudes higher than the unexposed polymer network. Another stress-activated polymer network was obtained by functionalizing polymer backbones with cyclobutene. Ultrasonication allowed mechanochemical ring opening of cyclobutene, ultimately forming α,β-unsaturated ester polymers [75]. Furthermore, with sonication of mechanophores it is possible to alter known reaction pathways, usually obtained from light- or thermal-induced reactions [55], [76]. Hickenboth and Moore et al. showed that *cis* and *trans* isomers of 1,2-disubstituted benzocyclobutene underwent electrocyclic ring opening upon sonication, which solely yielded in the E,E-isomer. This outcome stands in contrast to the products obtained from light- or thermal-induced reaction, where the *cis* and *trans* isomers resulted in different products [77]. Although, only a limited number of selective reaction pathways, induced by ultrasonic waves, are currently available, conducting further research is crucial to discover additional examples and comprehensively unravel the underlying mechanisms. Incorporating mechanosensitive units within the polymer network is a unique tool to strengthen polymeric materials under typically destructive forces. This important feature shows great potential for industrial applications as it can be used to engineer self-reporting and self-healing materials.

### 2.3 Sonocrystallization

Crystallization comprises two phenomena: nucleation and crystal growth. Furthermore, this process can be classified as either primary or secondary nucleation. Primary nucleation can be divided into either homogeneous or heterogeneous nucleation. The latter describes the presence of a solid interface, which does not influence the crystallization process. Whereas homogenous nucleation occurs in the absence of any solid interface so that high levels of supersaturation are required to induce nucleation. Secondary nucleation defines the situation in which the presence of crystals acts as templates for new crystal growth by contact nucleation. The resulting crystals can be influenced by various conditions during the reaction, such as temperature, pressure, and concentration [78]. Especially US is a useful tool to tailor desired properties of crystalline products [79]. Sonication can for example induce primary nucleation or it can generate fragmentations of existing crystals that further induce secondary nucleation [80], [81], [82]. Furthermore, ultrasonic waves are able to decrease induction time, nucleation rate, and metastable zone width [83]. In general, US enables the generation of smaller crystals with a decreased particle size distribution. As a result, sonication shows to provide control over the size, morphology, and purity of the desired crystals and prevents agglomeration of particles [83], [84]. Not only the control over the physiochemical characteristics upon ultrasonication is advantageous, but also the formation counts as energy and cost-effective, as it prevents harsh reaction conditions, such as long reaction times, high levels of supersaturation, high pressure and temperature [85]. Borse et al. were able to generate metal-organic frameworks via an environmentally friendly approach using ultrasonic waves. In particular, they report the first generation of nickel-doped zeolitic imidazole frameworks within waterborne polyurethane (WPU) [86]. High crystallinity with uniform size distributions of NPs were achieved after the solution was exposed to ultrasonic waves. The resulting nanofiller showed improved thermal and mechanical properties comparted to pristine WPU. Furthermore, increased NP loadings within WPU showed increased antibacterial activities against *E.coli* and *S.aureus* [86].

Not only does US help to create green crystallization pathways, but it can also be used to alter the resulting crystal properties. This was particularly helpful when sonocrystallizing paracetamol. Various research groups reported that ultrasonic waves influenced the crystallization process and the resulting crystal properties of paracetamol [82], [87], [88], [89]. Bučar et al. were able to improve the compaction behaviour of the monoclinic form of paracetamol by exposing the solution to low frequency US. This is especially of relevance for industrial purpose as the monoclinic, macrometer-sized form shows poor tabletability. Therefore, paracetamol tablets require the use of excipients, e.g. film coated hydroxypropylcellulose, to improve their tabletability [90]. However, US-assisted crystallization allowed the formation of nano- and micrometer-sized paracetamol crystals, which showed significantly higher elastic moduli and bulk cohesion. This ultimately resulted in better tabletability [89]. Guo et al. investigated the influence of roxithromycin in solvent free conditions under ultrasonication. Not only the induction time of the primary nucleation decreased significantly when exposing roxithromycin to ultrasonic waves, but also agglomeration of the crystals decreased. Furthermore, the nucleation rate increased, and sonication narrowed the metastable zone width, and induced nucleation at much lower levels of supersaturation where spontaneous primary nucleation cannot occur. Ultimately, ultrasonication influenced the morphology of the crystals from hexagonal to rhombus shape and the resulting crystals showed narrower particle size distributions [91].

**Fig. 5:**
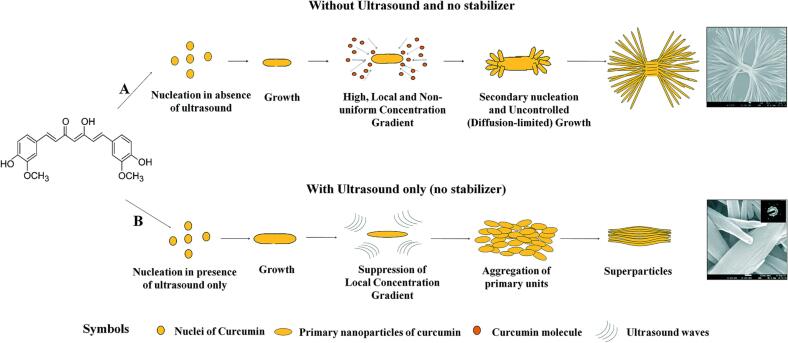
Crystallization process of curcumin (A) without US (B) with US. Reproduced from reference [92] with permission from The Royal Society of Chemistry.

Another example of influencing the morphology upon sonocrystallization is reported by Thorat and Dalvi. They investigated the growth rate, morphology, particle formation pathway, and crystal polymorphism of curcumin with and without ultrasonication [92]. In the absence of ultrasonication, micromixing is prevented and thus, high concentration gradients are observed. Therefore, diffusion-limited crystal growth kinetics are observed and led to uncontrolled dendrite formation (Fig. 5A). Ultrasonication increases mass diffusivity of curcumin in aqueous solution and leads to micromixing. Both phenomena are responsible for uniform supersaturation with low concentration gradients. Therefore, integration-controlled growth of curcumin crystals was observed, ultimately leading to smooth, uniform curcumin particles (Fig. 5B). Furthermore, orthorhombic curcumin was formed when ultrasonic waves have been applied during the crystallization, whereas absence of US led to the monoclinic form.

The previously highlighted examples serve to emphasize the compelling need for a more extensive investigation of the profound impact of ultrasonic waves on the process of crystallization. Exposure of known crystallization processes to US holds the potential to engineer versatile crystals with desired physiochemical properties. Notably, ultrasonic waves have demonstrated to enhance control over crystal size and morphology. Furthermore, a deeper understanding of the influence of ultrasonic waves has the capacity to overcome existing hurdles, including challenges related to nucleation control and the rate of crystal growth.

### 2.4 Sonocatalysis

Via wastewater treatment plants persistent organic pollutants, such as textile dyes or antibiotics, can be removed. Especially, advanced oxidation processes (AOPs) are used to efficiently degrade those compounds by oxidizing the pollutants with hydroxyl radicals obtained from for example ozone, hydrogen peroxide, UV light, and US. AOPs based on UV light and US are advantageous over the other procedures as they do not require addition of external chemicals. However, AOPs based on UV light, require long reaction times and the effectiveness of several water pollutants is limited [93]. Similar limitations are found with solely using US for AOPs as more than 50 % of the energy is converted in thermal dissipation. Therefore, high energy consumption is required to generate radicals which are involved in the AOP [94].

Especially, the so called sonocatalysis is a promising tool for wastewater treatments plants, where US is applied with the presence of heterogeneous catalysts [95]. In this way, not only the reaction time can be reduced, but also milder physical conditions allow the generation of radicals. This approach ultimately leads to a more environmentally friendly process. In general, the presence of catalysts upon ultrasonication increases the active sites for cavitation phenomena, which further increases the quantity of reactive radicals. Usually, an increase in frequency results in higher amounts of radicals [96]. When focusing on water decontamination, sonocatalysis is used for sonolytic degradation, which involves •OH, •H, and •HO2 radicals. Those radicals generally recombine to produce H_2_O. There are three underlying mechanisms that are responsible for the sonocatalytic performance.

(1) Thermal catalytic mechanism: This mechanism is based on the so called hot-spot theory, which has been discussed in the first chapter of this review. These hot spots are able to thermally excite semiconductors, which ultimately enable the generation of electron-hole pairs. These electron-hole pairs are responsible for •OH radical production, which further catalyse the desired reaction. In particular TiO_2_ NPs are well known sonocatalytic semiconductors, however, fast recombination of electron-holes limits the radical production [97], [98], [99]. The overall radical formation increases when preventing the electron-hole recombination ultimately, increasing the efficiency of the semiconductor. In particular, making use of co-catalysts, such as Au and Pt, can be helpful, as it hinders the recombination of electron-holes [100], [101], [102]. Kawamura et al. were able to almost double the •OH radical production when depositing Au_144_ nanoclusters on TiO_2_ [103].
(2) Photocatalytic mechanism: Similar to the thermal catalytic mechanism, the photocatalytic mechanism is based on the bubble cavitation phenomena upon ultrasonication in the presence of a semiconductor. In contrast to the previous one, bubble cavitation in the photocatalytic mechanism results in emission of light, which is also called sonoluminsescence. The resulting photon is able to excite electrons from the valence band to the conduction band, in which electron holes in the valence band are generated. The resulting exited electron can react with available oxidants to form reactive radicals, which further induce the desired reaction.
(3) Heterogeneous nucleation: This involves the presence of solid particles that provide preferential sites for acoustic bubble cavitation at the solid surface or nucleation site to generate •OH radical production. Again, free radicals are responsible to further induce the desired reaction. Heterogeneous nucleation can be influenced by either the reaction conditions (such as frequency, and power of an ultrasonic device, temperature), or the physiochemical properties of the solid particle (such as particle size, shape, pore size, and hydrophobicity) [93], [94], [104], [105], [106].

Previously, it has been shown that ZnO NPs are useful catalysts for the degradation of organic pollutants in waste water [107], [108]. Modifying the surface of ZnO NPs with Ag narrowed the band gap of the catalyst, and resulted in prolonging its lifetime, and thus, increased the overall sonocatalytic performance [109], [110]. Chan et al. showed that incorporation of Ag into the ZnO lattice increased the surface area and decreased the crystallite site of the resulting NPs. Furthermore, silver-doped ZnO NPs enabled 98 % of sonocatalytic degradation after 30 min ultrasonication of malachite green, a dye that is often used in the textile industry [110].

**Fig. 6:**
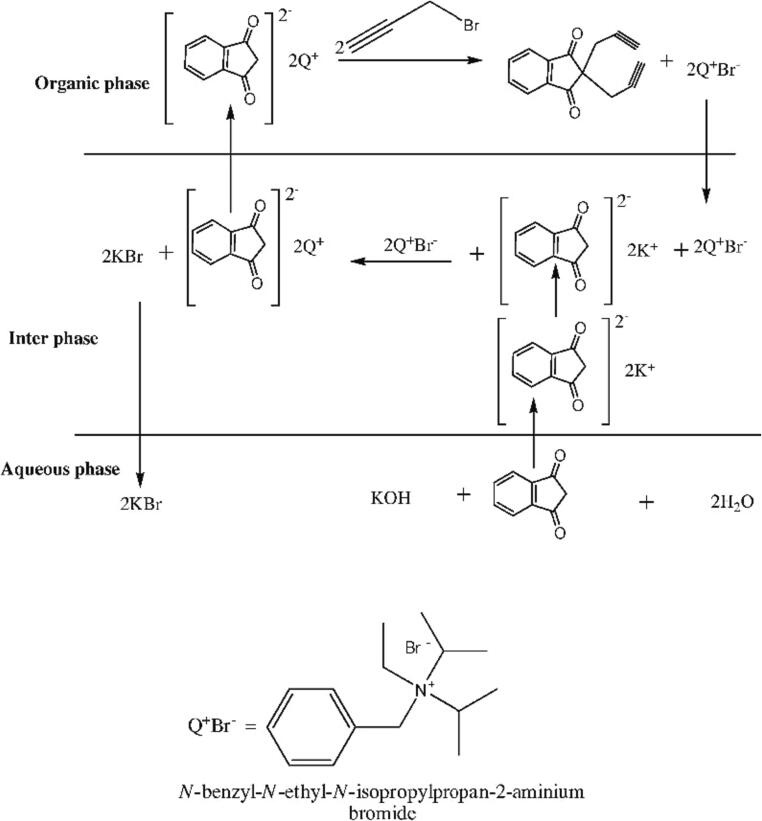
Proposed reaction mechanism of propargylation of indene-1,3-dione with propargyl bromide to produce 1,1-di(prop-2-ynyl)-1H-indene-1,3(2H)-dione in the presence of PTC and potassium hydroxide. Reproduced from reference [118] with permission from Elsevier.

Not only is sonocatalysis important for waste water treatment plants, it is also a very useful tool in organic synthesis, such as Diels-Alder reactions, Mannich reactions, Suzuki cross-coupling reactions, Claisen-Schmidt condensations, or Michael additions [111], [112], [113], [114], [115]. For the latter, Martin-Arander et al. showed, that ultrasonication promoted the Michael addition between imidazole and ethyl acrylate using alkaline clays in terms of the reactivity, and yield. Various reaction conditions with two different alkaline clays, namely Li^+^- and Cs^+^-montmorillonites, have been investigated with and without ultrasonic exposure. Treating the reaction for 15 min with ultrasonic waves at a temperature of 303 K with Cs^+^-montmorillonites more than doubled the yield in product, namely from 17.9 % to 37.0 %, compared to the non-ultrasonic treated reaction [115]. As previously discussed, US is a helpful tool to generate radicals in aqueous medium, promoting the overall reaction in terms of reaction rate, yield, and selectivity. However, in organic synthesis, most reactants and catalysts are not soluble in water, which limits the use of sonocatalysis for organic synthesis as aqueous solutions are required in order to generate hydroxyl radicals. In particular phase transfer catalysts (PTC) can overcome this limitation, as those catalysts are able to promote reactions between immiscible reactants and thus, enabling the transfer of reactants from one phase to the other [116], [117], [118]. In this way, US can still be applied for reactions in which water-soluble reactants and organic solvents are being used. In particular quaternary ammonium salts are often used as PTCs, such as tetrabutylammonium bromide (TBAB). Diwathe et al. sonocatalyzed 1-benzyloy-4-nictrobenzene from 1-chloronitrobenzene using TBAB as PTC in the presence of potassium hydroxide flaxes [119]. They investigated several parameters (e.g., temperature, stirring speed, solvent volume, concentration of PTC, ultrasonic power) and were able to show that an increase in the concentration of TBAB led to an increase in the reaction rate. Similar findings were shown for an increase in power of the US. Furthermore, using US-assisted PTC resulted in higher yields than the conventional non-ultrasonic method [119]. Various US-assisted PTC have been investigated. Selvaraj et al. synthesized a new quaternary ammonium salt PTC that promotes the propargylation of indene-1,3-dione [118]. For the *N*-benzyl-*N*-ethyl-*N*-isopropylpropan-2-aminium-bromide synthesis, which is used as the PTC, they reacted *N*-ethyl-*N*-isopropylpropan-2-amine with benzyl bromide and ethanol. Afterwards, Indene-1,3-dione was reacted with propargyl bromide under the presence of the previously synthesized PTC and chlorobenzene. Ultrasonication increased the overall reaction rate, selectivity, and yield compared to the non-irradiated reaction. The proposed reaction mechanism is displayed in Fig. 6, showing that the reaction takes place in the interphase of both immiscible solvents. Especially with the help of PTC this reaction can overcome the previously mentioned limitations of sonocatalysis in organic synthesis.

### 2.5 Sono-processes for surface modification

Another useful tool of ultrasonic waves is the chemical modification of surfaces upon exposure. US-induced surface modification has a wide range of applications and includes the functionalization or activation of surfaces, surface cleaning, surface coating, and NP synthesis. The underlying mechanisms varies depending on the application and materials being used. This subsection will focus on the most intrinsic findings for the use of US in the modification of surfaces.

#### 2.5.1 Surface coating

US is a widely used tool to create coatings with improved homogeneity and a consistent thickness. The underlying mechanism is based on US-induced microjets near the solid surface that are created after the cavitation of bubbles in liquid medium. These microjets are able to accelerate NPs up to a speed of 200 m/s, enabling the NPs to impinge on the solid surface, causing the NPs in the coating solution to penetrate into the solid surface [120]. Eshed et al. were able to homogeneously coat ZnO and CuO NPs using US irradiation on artificial acryl tooth implants. ZnO and CuO NP coated tooth implants had a thickness of 150 and 30 nm, respectively, and showed a reduction of 75 % and 85 % of *streptococcus mutans* biofilm formation compared to the non-coated one [120]. Also, Gottesman et al. were able to synthesize antimicrobial coatings. In this work they used Ag NPs that were ultrasonically coated onto parchment paper, in which the NPs penetrated the paper by more than 1 µm, resulting in highly stable coatings with antibacterial activity against *E.coli* and *S. aureus* [121]. Not only Ag coated paper shows potential for industrial applications, but also antibacterial textile fabrics show advantageous features for several applications, such as medical bandages, and wound dressing. Perelshtein et al. reported antibacterial activity of textile fabrics, such as nylon, polyester, and cotton, when sonochemically coated with Ag NPs [122]. This one-step reaction resulted in a uniform coating of silver NPs on the corresponding substrate and showed for all three textiles antibacterial properties against *E.coli* and *S.aureus.* Further examples of various NP-coated textiles with antibacterial properties using US irradiation have been established [123], [124], [125].

**Fig. 7:**
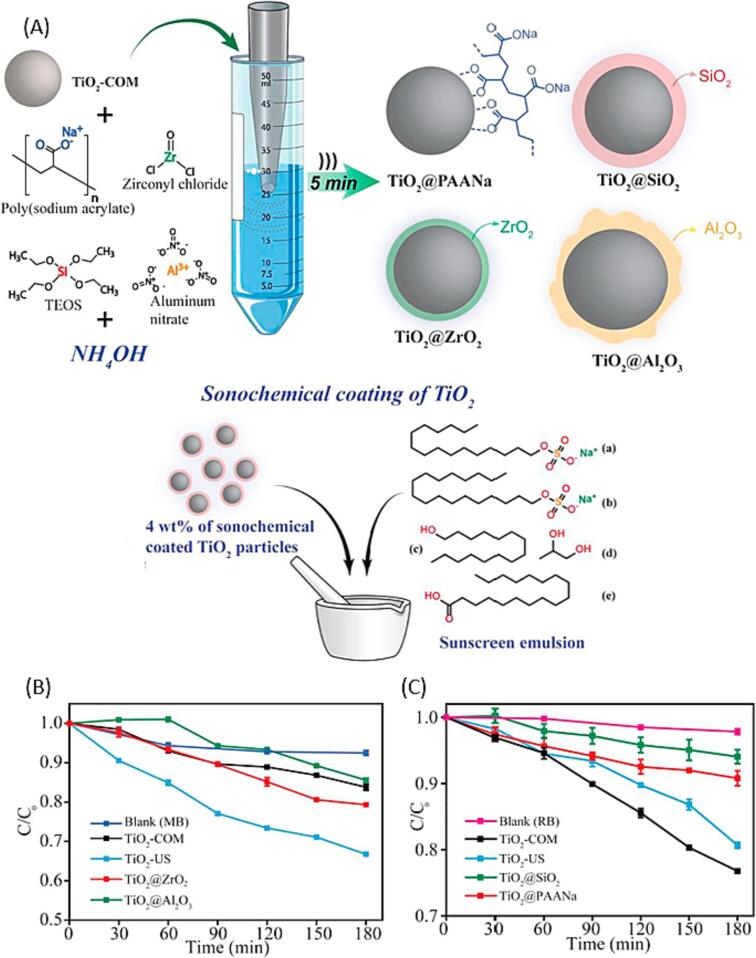
(a) Commercially available TiO_2_ NPs for sunscreen formulations ultrasonically coated with SiO_2_, Al_2_O_3_, ZrO_2_, and sodium polyacrylate and sonochemically coated TiO_2_ NPs mixed with sunscreen emulsions to test photocatalyic activity, colloidal properties, and sunscreen protection factor (SPF); Photocatalytic activity of (b) commercial TiO_2_ (TiO_2_-COM), TiO_2_-US, TiO_2_@ZrO_2_, TiO_2_@Al_2_O_3_ using Methylene Blue (MB) and (c) TiO_2_-COM, TiO_2_-US, TiO_2_@SiO_2_, TiO_2_@PAANa using Rohdamine B (RB). Adjusted with permission from reference [126]. Copyright 2018 Elsevier.

US can also be used to increase the photocatalytic activity of TiO_2_ NPs, which are commonly used in sunscreen formulations for protection against UV-radiation. Even though, TiO_2_ NPs in the size range of <200 nm show higher UV attenuation, they are often not used in commercially available formulations, as they have the tendency to form agglomerations. Barbosa et al. was able to overcome this limitation by coating TiO_2_ NPs with four different substrates (SiO_2_, Al_2_O_3_, ZrO_2_, and sodium polyacrylate (PAANa)) using ultrasonic irradiation (Fig. 7a) [126]. Ultimately, the coated NPs were combined with sunscreen emulsion and tested against their photocatalytic activity (Fig. 7b), colloidal properties, and sunscreen protection factor. Especially, the sodium PAANa coated TiO_2_ showed promising properties for sunscreen formulations as it showed similar properties for the Sunscreen Protection Factor (SPF), while the colloidal stability improved, and the photocatalytic activity decreased (Fig. 7c).

#### 2.5.2 Sonophysical effect for surface cleaning

Exposure of surfaces to ultrasonic waves is a widely used technique to clean surfaces, not only for research purposes, but also in industry. The underlying mechanism of surface cleaning when exposed to ultrasonic waves is based on the oscillation and cavitation of bubbles, heat, shear forces, and acoustic streaming near the substrate [127], [128]. However, Kim et al. showed that the main driving force for acoustic cleaning is based on the oscillation and cavitation of bubbles, as the other forces were too weak to detach microparticles from the substrate of interest [129]. Most often, acoustic cleaning uses low frequencies in the range of 20–40 kHz. However, it has been shown that the ultrasonic parameters, such as the frequency, and intensity, play a crucial role in the cleaning outcome. Kobayashi et al. investigated the cleaning performance of membranes when exposed to ultrasonic waves using three different frequencies (28 kHz, 45 kHz, and 100 kHz) and obtained best results for the lowest frequency [130]. This example shows how important it is to carefully consider the parameters during ultrasonic cleaning to minimize potential damage to the substrate. This is especially crucial in semiconductor industry where the damage to the surface must be minimized, and removal of nanosized particles, which is especially challenging, have to be successful. For these purposes, megasonic cleaning was developed, which is a cleaning procedure using high frequency ultrasonic waves, typically in the range of 0.8–2 MHz [131], [132], [133]. It is believed that megasonic cleaning results in a significant increase of the acoustic streaming when comparing to low frequency acoustic cleaning. The acoustic streaming decreases the adhesion force of dirt particles on the substrate, also allowing the removal of nanosized particles, ultimately resulting in clean surfaces [134].

Nowadays, acoustic cleaning is performed in a wide range of applications, from oil removal from oily sludges, to pesticide removal on agricultural plants, and cleaning solar panels [135], [136], [137]. However, most frequent use of acoustic cleaning is the removal or prevention of biofilm formation. This technique is especially helpful for medical implants, such as titanium implants used in dentistry, and indwelling medical devices such as urinary catheters [138], [139]. Not only is the biofilm formation in medical implants problematic, also biofilm formation on the hulls of ships has a tremendous environmental and economic impact as biofilms create a significant increase in friction, and thus, in fuel consumption. For this matter, removal of biofilms attached to antifouling coatings is crucial, where acoustic cleaning has shown to be a promising candidate [140], [141], [142].

Understanding the influence of each parameter during acoustic cleaning is important as ultrasonic waves can also be used for a combination of cleaning and nanostructuring the surface. The latter often happens unintentional, thus, knowledge about the possible outcomes is crucial. Especially, for nanostructuring the surface, properties of the corresponding characteristics of the substrate play an essential role. Skorb and Möhwald previously elaborated in detail the influence of US on nanostructuring the surface [143].

#### 2.5.3 Surface functionalization and activation

**Fig. 8:**
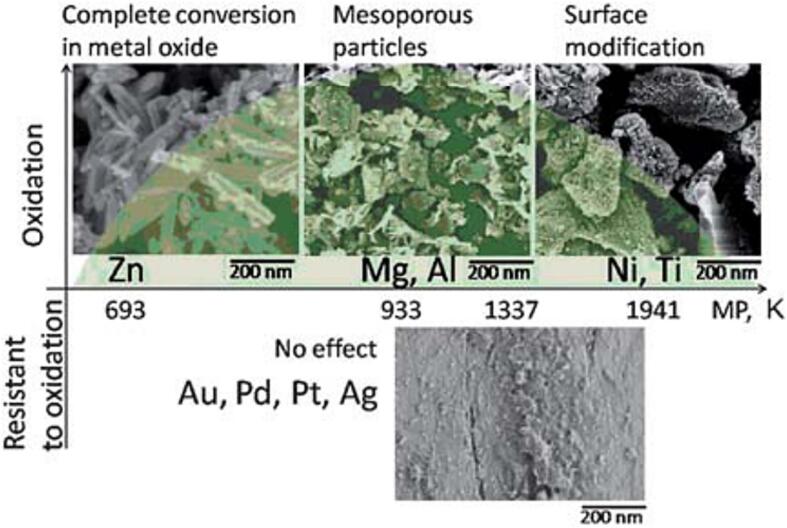
Ultrasonic exposure of various metals in which no effect was observed for metals resistant to oxidation and modification of metal particles, which are able to oxidize, was dependent on their melting point. Reproduced with permission of [145]. Copyright 2010 The Royal Society of Chemistry.

As shortly mentioned in the previous section, US is able to functionalize or modify the surface of the substrate of interest. Not only the reaction parameters, such as the temperature of the solution, exposure time, frequency, and intensity of the ultrasonic waves, are influencing the outcome, also the characteristics of the surface play an essential role. In this regard, the physical properties, such as stiffness, melting temperature, and polarity of the substrate, have a significant influence on the response to ultrasonic waves and the accompanying surface modification [143]. Sonochemical surface functionalization has been applied to a variety of substrates, including polymers, graphene, ceramics, and metals [144], [145], [146], [147]. For the latter, the characteristics of their reactivity, and melting points play an important role. Skorb et al. investigated the transformation of various metals when exposed to acoustic cavitation (Fig. 8). Under the same reaction conditions, no effect of acoustic cavitation in aqueous medium is seen for noble metals, such as Au, Pd, Ag and Pt, whereas, surface modification of Zn, Mg, Al, Ni, and Ti is obtained when exposing them to US [146]. For the non-noble metals, the corresponding melting point affected the transformation, in which complete conversion in metal oxide was seen for low melting point metals (Zn), whereas Mg and Al with moderate melting points turned into mesoporous particles, and Ni and Ti with high melting points, surface modification was observed.

Mason et al. conducted a study on the surface modification of Noryl HM4025, which is a blend of glass-filled polyphenylene ester and polystyrene. The strength of this research lies in its exploration of how various ultrasonic frequencies impact the surface modification by subjecting the material to an ultrasonic bath using five different frequencies [148]. Their findings revealed that higher frequencies corresponded to reduced weight loss of the substrate and less prominent surface modification. These results align well with the previously observed phenomenon of megasonic cleaning.

#### 2.5.4 Nanoparticle synthesis

A widely known field of the use of US is for the synthesis of NPs. The range of applications is innumerable and ranges from metal/magnetic NPs for the use in for example biomedicine [149], [150] or magnetic data storage [151] to semiconductor NPs, such as CdSe for photovoltaics [152], [153]. This research field has been extensively studied and has been exploited in various review articles already, therefore, only a brief discussion of this topic will be provided here [154], [155]. As already highlighted within the other sections of sonochemistry, the reaction conditions, such as solvent [156], [157], temperature [158], [159], reaction time [160], concentration [161], frequency [162], and intensity [163] of US, highly influence the properties of the resulting NPs, such as the size [164] and morphology [165]. Not only the high control over the size, composition, and morphology of the resulting NPs is advantageous, but also the short reaction time, low energy consumption, and easy scale-up of the synthesis, make US an attractive tool for NP synthesis [166]. A recent study by Chytrosz-Wrobel et al. demonstrated that US can facilitate the sonochemical generation of fluorouracil, an anti-cancer drug, as a NP drug carrier for potential controlled drug delivery. The researchers used molecular dynamics simulations to explain the mechanism underlying the NP formation at the molecular level and revealed that bubble cavitation plays a critical role in the formation of NPs’ seeds [167]. Despite the numerous promising studies on NP synthesis utilizing sonochemistry, one potential drawback of this approach is the generation of free radicals due to acoustic cavitation, which may lead to undesired side reactions.

## 3 US and its use in industry

### 3.1 (Bio-)medical field

One application of ultrasonic waves in the (bio-)medical field is ultrasonic cleaning by making use of bubble cavitation and acoustic streaming, which effectively remove contaminants and bacteria from the substrate’s surface [168]. However, it is important to note that not every material can withstand ultrasonic waves for cleaning or sterilization purposes, as it may lead to mechanical damage of the medical device [169]. Nonetheless, ultrasonic waves have diverse applications in the (bio-)medical field, including ultrasonic imaging, which has become crucial for diagnostic purposes. Furthermore, US has garnered attention for its use in sonodynamic therapy (SDT) and drug delivery systems, which will be further discussed in the following section.

#### 3.1.1 Ultrasonic imaging

Probably the most famous application of US, is ultrasonic imaging for medical purposes. Nowadays, ultrasonic imaging is one of the most used techniques for medical imaging. It has many advantages as it is inexpensive, portable, non-invasive, harmless, images in real-time, and is able to image cross-sections of the human body [170]. Ultrasonic imaging uses high frequencies sound waves in the range of 2–15 MHz to create images of the internal structures of the body. It is based on the principles of acoustic waves: propagation, refraction, reflection, and scattering [171]. Depending on the elasticity and density of the tissue, the acoustic impedance, the speed and direction of the waves, are influenced. This ultimately allows the distinction of different tissues [172]. Ultrasonic imaging is used in a broad field of diagnostics to identify abnormalities or lesions and is commonly used for various medical conditions such as prenatal tests, gall or kidney stones, heart diseases, and cancer diagnosis, such as breast or prostate cancer [173], [174], [175], [176]. However, the resolution and differentiation of different types of tissue, especially of areas with low blood flow, such as small vessels, are challenging to image with ultrasonic waves [177]. Particularly ultrasonic contrast agents (CAs) can help to overcome this limitation. CAs are gas filled bubbles in the micro- to nanometer range and are stabilized by either lipids, polymers, silica, surfactants, or proteins. Usually, the core of the bubbles consists of perfluorocarbons (PFC), such as perfluorohexane, or octafluoropropane, as they show poor solubility in blood and are biodegradable [178]. When exposing CAs to acoustic waves, the bubbles start oscillating in a nonlinear fashion [179], [180]. The reflection of the acoustic behavior of the MBs can be detected by the ultrasonic transducer, which increases the overall signal, and thus, enhances the signal-to-noise ratio of the US image, ultimately improving the contrast of the resulting image. Depending on the properties of the bubbles, such as the size and shell, the resulting acoustic behavior of ultrasonic CAs can be modified. Depending on the area of interest, the composition of the CA varies. Imaging for example cardiac vessels requires CAs that are able to withstand the mechanical stress of the cardiac cycle, while imaging a liver requires a higher half-life time of the MBs. The required properties can be adjusted by influencing the stability and stiffness of the shell of the ultrasonic CA. In general, CAs can be divided into two classes: soft- and hard-shell MBs. The latter are often stabilized using either polymers [181] or proteins [182], while soft-shell MBs usually consist of lipids, such as phospholipids [183]. Stabilizing the gas-liquid interface is crucial as the MB would otherwise dissolve nearly instantaneously. Thus, the stabilizing agents allow to control the physical properties of the resulting MB, but also increase the MB’s overall lifetime. The stability of a MB is negatively affected by the so-called Laplace pressure [184].

$$\Delta P=P_{b}-P_{a}=\frac{2\sigma }{r}$$

where P_b_ is the pressure inside the MB, P_a_ is the surrounding pressure, σ is the surface tension of the bubble, and r its radius. When introducing CAs into the bloodstream, their maximum size is restricted since it cannot surpass the size of pulmonary capillaries [185]. Additionally, the resonance frequency, on which the MBs resonate, is dependent on their radius. Medical US typically operates at frequencies ranging from 2 to 12 MHz, requiring MB radii in the range of 1–10 µm in diameter [186], [187]. The small size of MBs however, increases the Laplace pressure, making it essential to select an appropriate surface stabilizer to decrease surface tension and ultimately produce stable MBs. At present, there exist multiple MB-based CAs that are employed in clinical settings, including SonoVue®, Lumason®, Definity® and Optison® [188], [189]. Their applications range from amplifying the echogenicity of diverse organs such as the liver and kidney, to improving the visualization of the heart chambers or bloodstream [185], [190]. Typically, these CAs have a duration of action lasting around 5–10 min. Overall, these CAs have significantly improved the diagnostic capabilities of medical US [191]. Nonetheless, future CAs can be optimized in view of the brief lifespan of these agents as it constrains the duration of imaging procedures. Moreover, the restricted tissue penetrability of MBs, for instance in fatty tissues or bones, restricts their effectiveness for use in such applications.

#### 3.1.2 Sonodynamic therapy (SDT)

**Fig. 9:**
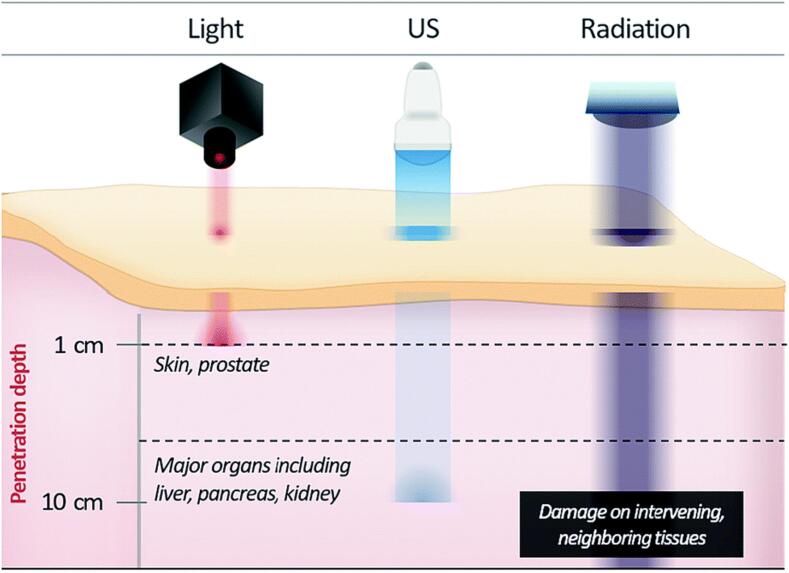
Schematic representation of penetration of different energy sources: (a) light used for PDT, (b) US used for SDT, and (c) Radiation used for various cancer treatment. (a) has very limited penetration depth for human tissue restricting the use of PDT for superficial cancer treatments, such as skin cancer, (b) shows an increase in penetration depth allowing the treatment of major organs, such as liver or pancreas cancer, and (c) shows high penetration depth, allowing the treatment of deeper tumor sites, however radiation damages intervening and neighboring tissues. Adapted and reproduced from [192] with permission of The Royal Society of Chemistry.

Photodynamic therapy (PDT) is a well-known approach for cancer treatment, in which a photosensitive drug is activated with light. However, the penetration depth of light is restricted, and therefore, PDT is only applicable for light-exposed regions, limiting the amount of tumor cells that can be treated (Fig. 9a). SDT can overcome this limitation, as US is able to penetrate deeper into tissue (Fig. 9b) [192]. The underlying principle is similar to the one of photodynamic therapy. However, instead of using a light source together with photosensitive drugs, SDT is based on an US probe combined with US sensitive drugs, so called ‘sensitizer’ drugs [193]. Upon ultrasonication, the drug is being activated via bubble cavitation, and the drug generates reactive oxygen species (ROS), which can damage, for example, lipid membranes, DNA, and RNA, ultimately causing apoptosis of the target tumor cells. Without US, the drugs show no cytotoxic effects. This phenomenon can also be explained with the quantum yield (QY), which defines the efficiency of the sonosensitizer by looking at the quantity of generated ROS [194], [195]. Thus, the higher the QY, the higher the efficacy of the resulting SDT. In contrast to US imaging, SDT uses focused, low intensity US, which ensures a tissue-selective activation. In this way, the activation of drugs occurs on specific regions of the body, limiting the risk of harm to the surrounding healthy tissue. Prominent examples for sonosensitizers are xanthenes, porphyrins, and phthalocyanines [196]. However, it has been reported that current drugs used for SDT have a high clearance rate and therefore, show insufficient accumulation in tumor cells, ultimately limiting the ROS generation. A promising tool to overcome low QY is the use of nanomaterials, either by nanoencapsulating sonosensitizers, or combining sonosensitizers with NPs [192]. Not only can nanomaterials be used to increase the target specificity by tailoring the desired properties, such as the surface functionality, but they are also known to increase the overall efficiency of SDT as NPs can act as nucleation sites for the bubble cavitation [197]. Various nanomaterials have been reported to increase the overall QY, such as carbon nanotubes, liposomes, and NPs based on metal oxides, such as TiO_2_, and ZnO. Especially the latter NPs are useful, as they entrap electron-hole pairs, and thus, increase the overall ROS production [192]. Wang et al. coated Au and ZnO NPs onto graphene nanosheets, in which ZnO produces separated electron-hole pairs upon ultrasonication. These are further transferred to the Au NPs via the graphene nanosheets, ultimately inhibiting the recombination of electron-hole pairs, and thus increasing the generation of ROS. They were able to significantly increase the QY and kill various cancer cells, such as HeLa and CT26 [198].

Even though there are several advantages, such as deep penetration of acoustic waves and limited harm of US to human tissue when compared to radiation therapy (Fig. 9c), SDT is still in the early stage of research and more expertise is required to fully understand its mechanism. In particular, the limited ROS production, and thus, the low QY of SDT must be improved. Nonetheless, the research provided until this day shows that SDT is a promising technique for future non-invasive treatment options for various cancer diseases. Currently, there are several ongoing clinical trials, in the recruiting status, for which all of them are focusing on the treatment of glioma using SDT in combination with different sonosensitizers (NCT04845919, NCT05370508, NCT05362409, NCT04559685). Especially, treating glioma with SDT holds promising outcomes, given that low intensity focused US can effectively and non-invasively target deep-seated lesions.

#### 3.1.3 Drug delivery systems

Making use of the oscillation behavior of MBs when exposed to acoustic waves is not only useful for contrast enhancement for imaging purposes, but also shows great potential for drug delivery systems. There are four ways in which the drug can be incorporated into MBs: (1) encapsulation of the drug inside the core of the MB, (2) adsorption of the drug onto the surface of the MB, (3) conjugation of the drug via chemical attachment on the surface of the MB, or (4) coating the MB with a layer of drug-loaded material[199]. Furthermore, MBs can be specifically functionalized or conjugated with ligands, such as antibodies, peptides, or aptamers, that bind to receptors or antigens on the surface of the target cell. The so called ‘US-targeted microbubble destruction’ (UTMD) is a promising field of research as cavitation of the MBs enables the local release of the payload of the MBs, therefore minimizes off-target effects, improves drug delivery efficiency and reduces systemic toxicity [200], [201], [202]. Li et al. synthesized a liposome-MB system loaded with sinoporphyrin sodium, a sonosensitizer drug, to treat breast cancer. They showed improved results when combining the loaded liposome-MBs together with US treatment. In particular, in vitro studies showed enhanced cell apoptosis, mitochondrial damage and changes to cell morphology. Furthermore, *in vivo* studies using a 4T1 mouse mammary tumor model showed significant anticancer activity by inhibiting tumor growth and reduced proliferation activity [203]. Sinoporphyrin loaded MBs had an average size of 1 µm indicating favorable characteristics for undergoing oscillation upon exposure to medical US. Subsequent investigations should explore the potential utility of this system as US CAs. This dual functionality could enable concurrent imaging and therapeutic treatment.

Liu et al. were able to synthesize a dual targeting MB system, allowing real-time US imaging on the one hand and on the other target tumor gene therapy by functionalizing the MB surface with gene delivery agents [204]. They integrated PEI to obtain cationic characteristics on the surface of the MBs and subsequently conjugated either iRGD peptides or CCR2 antibodies or the combination of both for targeting strategies for breast cancer therapy. They loaded these MBs with plasmid DNA, ultimately enhancing the gene transfection efficiency, leading to tumor growth inhibition both in vitro and *in vivo*. Furthermore, MBs have gained significant interest as drug delivery systems in brain cancer therapy due to their ability to open the blood-brain barrier (BBB) when exposed to US, thereby enabling the drug to penetrate the barrier and reach the desired target. Zhao et al. created a system that utilizes DNA-loaded MBs to disrupt the BBB, ultimately, leading to the suppression of C6 glioma growth in rats when subjected to focused US [205]. However, only small animals are used as an *in vivo* model. When comparing their composition with the skull of humans, the required penetration depth and resulting attenuation of ultrasonic waves of the significantly bigger skull could potentially hinder the applicability of this system [206], [207]. For a comprehensive overview of the application of MBs in opening the BBB, a recent review by Kim et al. is then of interest [208].

Not only UTMD is a promising approach for drug delivery systems, but also NPs can be used as drug carriers due to their ability to improve drug solubility, stability and bioavailability. However, controlling drug release from NPs at a specific time and location remains a challenge. One promising approach is the use of US to trigger the drug release. Several types of NPs have been established up to date, including lipid-based NPs, polymer-based NPs, mesoporous silica NPs (MSNs), and gold NPs. MSNs have been extensively studied, as the resulting properties, such as size and morphology, can be easily modified by changing the reaction conditions, such as pH, and temperature [209]. Li et al. engineered a drug delivery system using MSN that exhibits dual responsiveness [210]. This system effectively delivers the anticancer drug doxorubicin (DOX) by responding to two different stimuli. Firstly, in a low pH environment, the system releases the drug, enabling targeted treatment. Secondly, triggered drug release occurs when subjected to high-intensity focused US (HIFU), due to ultrasonic cavitation. This furthermore enables a pulsatile release of the drug. However, these are only preliminary findings as in-vitro and *in vivo* studies are missing. More importantly, the settings of the applied HIFU have overlooked the crucial mechanical index (MI) parameter, which is essential in clinical settings to prevent biomechanical side effects of ultrasonic waves. The MI is calculated by dividing the peak rare fractional pressure by the applied frequency. According to FDA guidelines, the MI must remain below 1.9 [211]. Consequently, complying with the permissible MI could hinder the triggered DOX release when exposed to HIFU.

**Fig. 10:**
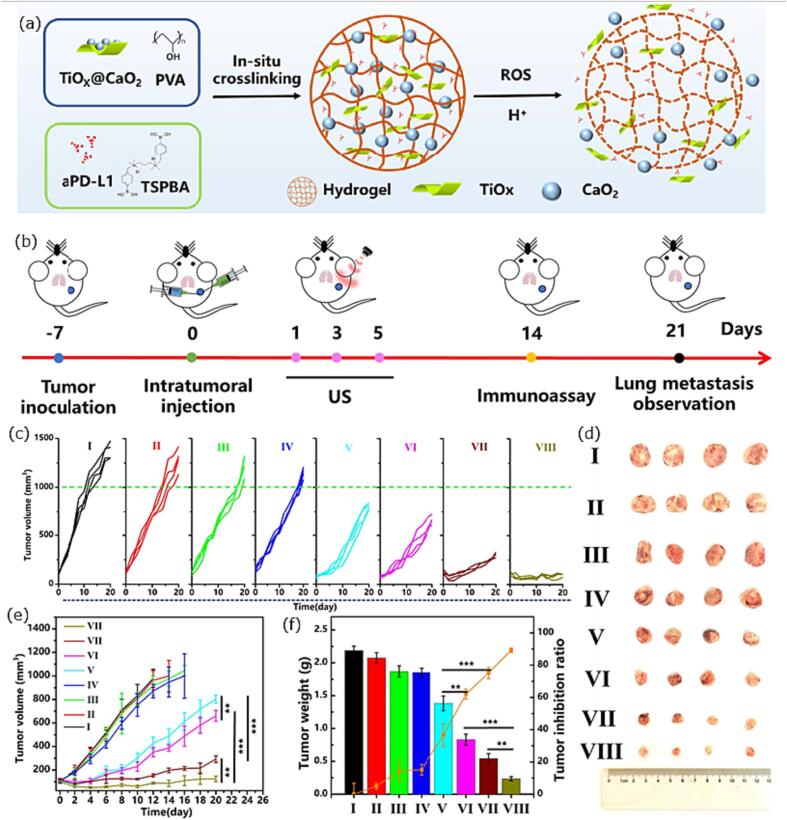
(a) schematic illustration of the dual hydrogel system, (b) schematic illustration of the treatment plan using a T41 mice model, (c) individual tumor growth, (d) photographs of tumor after treatment, (e) average tumor volume, (e) tumor volume, (f) tumor weight for all eight groups. Adjusted from reference [212] with permission of Elsevier.

Another system in which US triggered the release of drugs has been recently published by Dai et al [212]. They elaborated a dual hydrogel system (Fig. 10a), in which they combined the approach of SDT and chemodynamic therapy by incorporating a sonosensitizer (TiO_x_@CaO_2_) together with an immune checkpoint inhibitor using anti-PD-L1 antibodies (aPD-L1). In particular, they investigated the *in vivo* effect of the hydrogel in triple negative breast cancer using a 4T1 mice model (Fig. 10b). Upon ultrasonication an increased effect on the cell apoptosis could be observed due to two mechanisms: (1) CaO_2_ generates O_2_, which reduced the hypoxic tumor environment and further counteracts tumor immunosuppression, and (2) TiO_x_ forms OH· radicals, which activates ROS, enhancing cell apoptosis. Furthermore, the generation of Ca2+ ions damage the mitochondria, which result in an increased release of tumor antigens. Those antigens are tackled by aPD-L1 antibodies, ultimately resulting in good anti-tumor therapeutic effects. The research group divided the research in eight groups: (I) PBS, (II) US, (III) Hydrogel without sonosensitizer and immune checkpoint inhibitor, (IV) Hydrogel + aPD-Li, (V) Hydrogel + TiO_x_@CaO_2_, (VI) Hydrogel + TiO_x_@CaO_2_ + US, (VII) Hydrogel + aPD-Li + TiO_x_@CaO_2_, (VIII) Hydrogel + aPD-Li + TiO_x_@CaO_2_ + US, investigating the tumor growth. The combination of the dual hydrogel system with ultrasonic treatment showed the best results, in terms of tumor growth inhibition, tumor volume, and tumor weight (Fig. 10c – f). Additionally, there are other combined approaches using antibubbles, double emulsion templated MBs, where drug-loaded bubbles could be both visualized and destroyed by US and thereby deploying their payload at the location needed [213]. For a more detailed overview of drug delivery systems responding to ultrasonic waves the reviews of Zhao et al. and Low et al. are then of interest [207], [214].

### 3.2 State of development in the (bio-)medical field

In the previous section many examples of the use of US for the (bio-)medical field have been discussed and are highlighted in Table 1. CAs designed for medical US are accessible on the market and have been integrated into clinical practice to enhance the clarity of US images in difficult-to-scan areas, such as the liver or heart chambers [215]. However, the practical application of many other systems, such as SDT or drug delivery systems using US to mediate or trigger the release of the drug, in real clinical scenarios still presents challenges. Hereby, ensuring the safety of the patient and lowering toxic/side effects of sonosensitizers, and drug delivery systems, such as MBs, and NPs, remain the biggest hurdle [60]. Comparing the translation of research in the (bio-)medical field poses distinctive challenges that distinguish it from other industries. The (bio-)medical sector adheres to stringent regulatory standards to ensure the safety and efficacy of medical devices, drugs, and treatments. Furthermore, ethical considerations not only in the patient’s safety but also for conducting *in vivo* pre-clinical trials higher the burden of its translation. In the case of US responsive systems, a limited mechanical energy of the US wave and a specified frequency range, contribute to the complexity of designing a corresponding system [61]. Consequently, many preliminary studies, particularly in the field of drug delivery systems, are not applicable, as the required frequency or power output for initiating the drug release, frequently fall outside the medical range or pose potential risks to patient safety [216]. Despite the efforts, SDT and drug delivery systems are still in the pre-clinical state and more thorough clinical trials are required to establish standardized protocols including exposure time and US wave parameters to ultimately accomplish regulatory approval from health authorities [217]. This is a resource-intensive process which can only be achieved with collaborative efforts among researchers, clinicians, regulatory agencies and industry partners.

**Table 1:** Overview of state-of-art systems in (bio-)medical applications using ultrasonic waves.

Function Type	Working System	State of Development	Assessment	Source
CAs	SF_6_ filled MBs, stabilized by phospholipid distearoylphosphatidylcholine (DPPC) and polyethylene glycol (PEG 4000).	Commercialized as SonoVue®/Lumason®	High control over size distribution 99 % <10 µm with maximum size of 20 µm [220]	[188]
	C_3_F_8_ filled MB, stabilized by human albumin	Commercialized as Optison®	Broad size distribution 96 % <10 µm with maximum size of 32 µm [220]	[218]
	C_3_F_8_ filled MB, stabilized by phospholipid DPPC, dipalmitoylglycerophosphatidic acid (DPPA), and polyethylene glycol methyl ether (mPEG500)	Commercialized as Definity®/Luminity®	High control over size distribution 96 % <10 µm with maximum size of 20 µm [220]	[219]

SDT	Oral treatment with 5-ALA and subsequent exposure to US to treat glioma	Clinical Trial Phase 1+2	No results yet	Clinical Trials NCT05362409 NCT04845919
	Oral treatment with SonoALA-001 and subsequent exposure to MR-Guided Focused US to treat glioma	Clinical Trial Phase 1+2	No results yet	Clinical Trial NCT05370508
	Dual responsive hydrogel with incorporated sonosensitizer (TiO_x_@CaO_2_) and immune checkpoint inhibitor (aPD-L1)	Pre-clinical in-vivo study	Stimuli responsive sonosensitizer with incorporated target site for specific tumor delivery ultimately minimizing potential side effects and toxicity of surrounding healthy tissue	[212]
	Liposome based MB filled with synoporphyrin sodium as sonosensitizer to treat breast cancer	Pre-clinical in-vitro and in-vivo	Narrow size distribution of drug carrier; MBs are <10 µm, which is important for vascular perfusion when injected intravenously; Increased anti-cancer activity when combining sonosensitizer with US exposure	[203]

Gene delivery	Cationic MB conjugated with iRGD peptide and CCR2 antibodies to target tumor for breast cancer with simultaneous molecular imaging	Pre-clinical in-vitro and in-vivo	Tumor specific target due to surface modification with incorporated plasmid DNA for tumor gene therapy minimizing potential side effects	[204]
	glioma-and-neovascular-cell- targeting shRNA loaded MB against glioma	Pre-clinical in-vitro and in-vivo	Dual approach: MBs for BBB opening and target shRNA delivery	[205]

Drug delivery	DOX loaded polydopamine coated MSN for cancer treatment	Laboratory	HIFU did not include restriction of MI; Future research should further elaborate on clinical applicability including in-vitro and in-vivo experiments	[210]

### 3.3 US-assisted use in non–(bio-)medical industries

US, widely recognized for its diagnostic and imaging capabilities in the medical field, has increasingly demonstrated its versatility and effectiveness which has led to its adaption in various industrial sectors. This powerful technology has been harnessed to address numerous industrial challenges, offering green innovative solutions and enhancing various processes. Significant benefits for the use of US can be found for environmental applications [221]. Especially, exposing wastewater treatment plants to US has demonstrated significant improvements for the removal of various pollutants, such as organic dyes, antimicrobials, and chemical residues [39], [222], [223], [224], [225]. As mentioned in 2.4, US-assisted techniques can effectively degrade organic pollutants via AOPs. Ioannidi et al. recently showed that low frequency US enabled the removal of the drug dexamethasone from aqueous solutions [226]. They were able to eliminate 500 µg/l of the drug within less than 60 min. Furthermore, US can be applied to remove organic dyes, which are widely used in textile or cosmetic industry. It has been shown that Basic Red 46 was successfully degraded using a sonocatalytical approach by combining US with bentonite-supported ZnO NPs [227]. Furthermore, US shows great potential for oil-contaminated sand cleaning [228], soil remediation [229], [230], and algal bloom in aquatic ecosystems [231], contributing to sustainable practices for environmental applications by ensuring the protection and preservation of ecosystems and promoting cleaner and healthier environments [228]. The company Moleaer Inc. has specialized in the generation of nanobubbles that are injected into water to further enhance treatment processes [232]. This technology is mainly based on enhancing oxygen transfer and improving aeration efficiency. Thus, it can be applied in many industrial sectors reaching from agriculture by improving Standard Oxygen Transfer Efficiency, which significantly increases the harvest yield, to wastewater treatment plants or algae growth control in standing waters [233]. Another company that utilizes nanobubble technology is Cavitation Technology Inc., and finds applications in wastewater treatment plants, food industry for beverages and vegetable oil refinery.[234], [235], [236].

US finds crucial applications not only in environmental aspects for the removal of contaminations but also in food industry, such as microbial, mycotoxin, and fungi decontamination [237], [238]. Furthermore, US is an effective tool for food preservation, as high intensity US enables the deactivation of enzymes and microbial populations, extending the shelf life of perishable food items. For the latter, Sanovo offers the so called ‘SonoSteam Technology’, which specialized in the decontamination process for food preservation by using steam in combination with US [239]. Commonly, the food industry uses ultrasonic waves for processing milk and wine as it helps to maintain nutritional value, flavor and texture of the products while reducing the need for chemical additives or high temperature treatments [240]. Singla et al. provided a comprehensive overview of the applications of US in various aspects of food processing [241]. Their study highlighted the wide range of applications, including food extraction, emulsification, and filtration.

Furthermore, US and sono-processes play an important role in coating applications primarily attributed to their remarkable capacity to precisely regulate thickness and ensure uniformity. SonoTek Corporation stands as the global frontrunner in utilizing US technology for coating production [242]. This innovative approach finds diverse applications across industrial sectors, including semiconductor, automotive, and textile coating and exhibits promising solutions for nanomaterials, including fuel cells and solar cells [243].

Lastly, sono-processes play an important role in the energy industry, particularly in renewable energy sectors as US has a significant impact on the production and maintenance of photovoltaic cells and panels, aiding in cleaning [244], and defect detection [245], [246]. For the latter, Zetec has specialized in US-based non-destructive testing in many industrial sectors including inspections in the transportation sector such as train-rails, for the oil and gas sector for up-, mid-, and downstream solutions, and for the energy sector to detect damages on windmills [247]. Overall, sonochemistry contributes to the development of new materials and catalysts for energy storage and conversion systems, such as batteries and fuel cells [248], [249], [250]. Furthermore, US is also utilized for hydrogen production [251], [252], and optimization of biofuel production processes, improving extraction efficiency and enhancing the conversion of biomass to biofuels [253], [254], [255], [256].

## 4 Conclusion and discussion

In conclusion, sonochemistry offers significant potential for various chemical reactions, leveraging the effects of US to enhance reaction rates, alter chemical pathways, increase product yields, and operate under milder conditions, promoting energy efficiency and reducing the need for hazardous reagents. Additionally, sonochemistry allows for precise control over particle size and morphology, making it valuable in nanotechnology and materials science [164], [165]. The latest findings of sonochemistry have shown promising outcomes for various branches in chemistry, such as electrochemistry, organic synthesis, mechanochemistry, crystallization, its use for environmental remediation, and materials processing. However, these reactions are typically performed on a small scale, making it challenging to upscale the processes for industrial applications. Therefore, more research is necessary, emphasizing on the industrial translation. Additionally, further investigations are required to explore novel reaction mechanisms, thereby focusing on the selectivity of the reaction. Currently, ultrasonic waves can affect multiple chemical species simultaneously, leading in many cases to non-specific reactions and reduced selectivity, which ultimately limit the control over specific reaction pathways. Furthermore, sonication can generate heat and therefore affect the temperature of the reaction medium [257]. This characteristic can particularly impose limitations on its usage in temperature-sensitive reactions since elevated temperatures might cause the decomposition of reactants or catalysts. Additionally, sonochemistry is a new evolving field of research, and the selection of the operating frequency and power for ultrasonic waves is often not wisely chosen. More research is necessary to further elaborate on the impact of these parameters to ultimately improve the efficiency and selectivity of reactions.

So far, sonochemistry has found utility in various industrial sectors, including wastewater treatment, pharmaceuticals, food processing, and energy production. It has been employed for drug synthesis, extraction of bioactive compounds, emulsification, and enhancing the efficiency of chemical processes. Furthermore, its applications in sustainable chemistry, such as the degradation of pollutants, align with the growing demand for environmentally friendly processes [37], [38], [39], [40].

However, in order to further expand the scope of sonochemistry and pave the way for future application-driven advancements, fundamental inquiries need to be addressed:

• Which additional reaction mechanisms can be selectively altered by utilizing ultrasonic waves?
• What strategies can be implemented to optimize US systems to further enhance the energy efficiency of ultrasonic wave generation, thereby expanding its practical adoption in various industries?
• How can the scalability of current sonochemical reactions be improved?

Addressing these questions will increase the applicability of sonochemistry in a wide range of industrial sectors and holds promising prospects for further advancements.

When considering the use of sonochemistry in the (bio-)medical sector, a significant portion of the research remains in the laboratory phase. This is due to the sophisticated process of creating systems for medical applications, ultimately challenging the transition to market-ready products. The demands placed on such systems are notably greater compared to other industries [217]. Ultrasonic parameters, including frequency, power output, and duration, must adhere to medical standards. Furthermore, potential side effects of the system must align with regulatory guidelines, adding to the complexity of the development process. This challenge is in particularly pronounced in drug delivery systems as the required parameters to trigger the drug release or activate the drug upon ultrasonication often fall outside the medical applicable range [217]. Furthermore, *in vivo* models used in early stage research often do not have the same penetration depth and resulting attenuation of ultrasonic waves as in human bodies, which could ultimately hinder the applicability of those systems. To enhance the translation to clinical application, laboratory research should prioritize the system's potential for practical use even in its preliminary stages.

## Declaration of Competing Interest

The authors declare the following financial interests/personal relationships which may be considered as potential competing interests: Patrick van Rijn reports a relationship with BiomACS BV that includes: equity or stocks. P.v.R also is co-founder, scientific advisor, and share-holder of BiomACS BV, a biomedical oriented screening company. The authors declare no other competing interests. The authors declare no further conflict of interest.

## Data Availability

No data was used for the research described in the article.
